# Paths taken towards NK cell–mediated immunotherapy of human cancer—a personal reflection

**DOI:** 10.1111/sji.12993

**Published:** 2020-11-20

**Authors:** Hans‐Gustaf Ljunggren

**Affiliations:** ^1^ Department of Medicine Center for Infectious Medicine Karolinska Institutet Stockholm Sweden

**Keywords:** cancer, immunotherapy, MHC class I, missing‐self, NK cells, tumour immunity

## Abstract

The discovery that NK cells are able to specifically recognize cells lacking the expression of self‐MHC class I molecules provided the first insight into NK cell recognition of tumour cells. It started a flourishing field of NK cell research aimed at exploring the molecular nature of NK cell receptors involved in tumour cell recognition. While much of the important early work was conducted in murine experimental model systems, studies of human NK cells rapidly followed. Over the years, human NK cell research has swiftly progressed, aided by new detailed molecular information on human NK cell development, differentiation, molecular specificity, tissue heterogeneity and functional capacity. NK cells have also been studied in many different diseases aside from cancer, including viral diseases, autoimmunity, allergy and primary immunodeficiencies. These fields of research have all, indirectly or directly, provided further insights into NK cell‐mediated recognition of target cells and paved the way for the development of NK cell‐based immunotherapies for human cancer. Excitingly, NK cell‐based immunotherapy now opens up for novel strategies aimed towards treating malignant diseases, either alone or in combination with other drugs. Reviewed here are some personal reflections of select contributions leading up to the current state‐of‐the‐art in the field, with a particular emphasis on contributions from our own laboratory. This review is part of a series of articles on immunology in Scandinavia, published in conjunction with the 50th anniversary of the Scandinavian Society for Immunology.

**FOREWORD**

More than 35 years ago, just having turned 22‐years old, I began working as an undergraduate student in the Department of Tumor Biology at the Karolinska Institute, in the laboratory of Dr. Klas Kärre, a recent MD, PhD graduate. The goal of my summer project was to investigate whether certain immune cells (called NK cells) could recognize tumour cells lacking expression of specific “self” molecules, in the mouse referred to as H‐2. Highly polymorphic H‐2 molecules were known to be expressed on almost every cell in the body, distinguishing different strains of mice from each other. Never would I have envisaged, still being in the field almost four decades later being involved in the development of clinical NK cell‐based immunotherapies for human cancers. Here, I review some select findings from our laboratory that has contributed to present state of art regarding NK cells and NK cell‐based immunotherapies. This review is part of a series of articles commissioned by the Scandinavian Journal of Immunology in conjunction with the 50th anniversary of the Scandinavian Society for Immunology. The series covers key immunological developments in Scandinavia, or by Scandinavian scientists, over the last 50 years.

## 1 INTRODUCTION

In the early 1970s, studies by Kiessling et al^1^, ^2^ at Karolinska Institutet, in Stockholm, Sweden, led to the discovery of NK cells. Similar findings were reported in parallel by Herberman et al^3^, ^4^ at the NIH, in Bethesda, USA. NK cells were suggested to represent a specific type of cell of the immune system specialized in eliminating cancer cells. Soon afterwards, NK cells were found to also recognize virus‐infected cells.^5^ It was not understood how NK cells recognized target cells and experimental work was very much focussed on finding *the* NK cell receptor.^6^ Clearly, the field was biased by the recent discovery of the T cell receptor.^7^, ^8^ However, a common denominator of NK cell recognition of target cells was their ability to sense cells lacking expression of specific self‐molecules (very much in contrast to T cells). This and other published results had led Klas Kärre to hypothesize that NK cells might be triggered by target cells lacking expression of self‐specific antigens.^9^, ^10^

## 2 MHC CLASS I‐DEFICIENT CELL LINES REVEAL NK CELL RECOGNITION OF “MISSING‐SELF”

**FIGURE 1:**
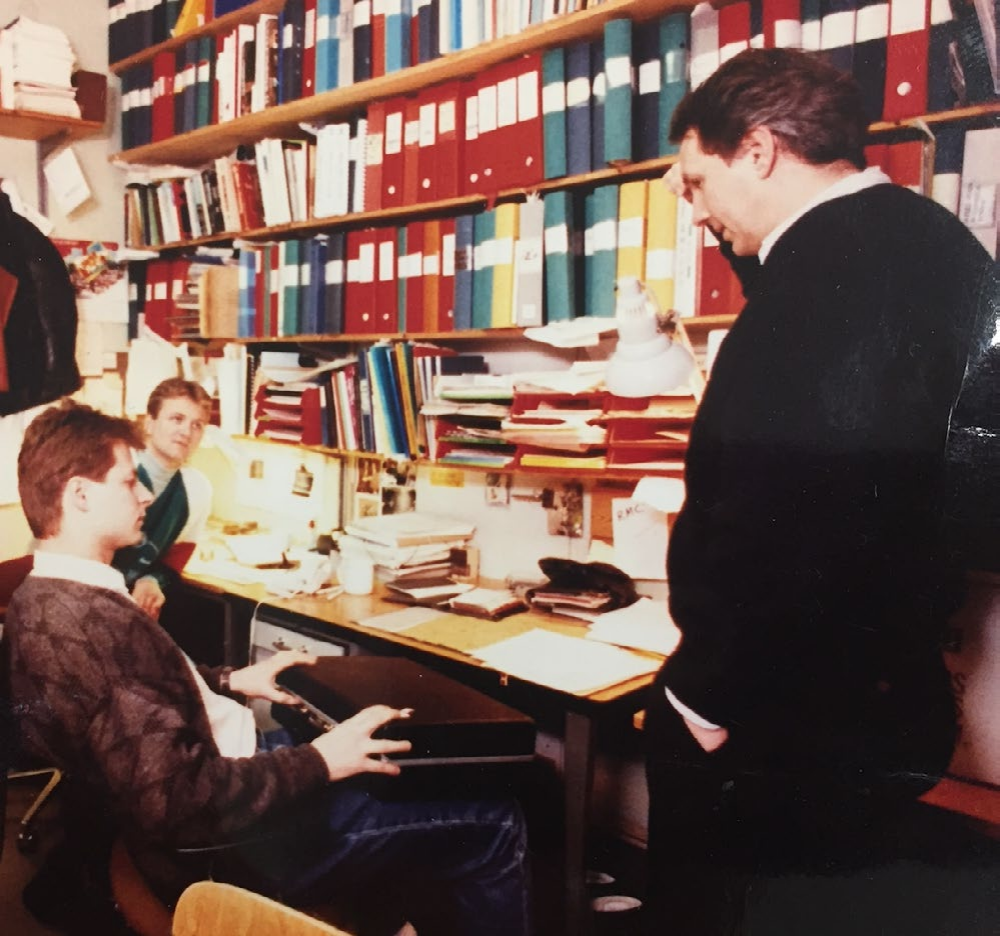
A day in the office at the Department of Tumor Biology. A day in the office at the Department of Tumor Biology, Karolinska Institutet, mid/late 1980s. In the far left, Petter Höglund, currently the Chairman of the Department of Medicine, Karolinska Institutet. In the right part of the picture, Klas Kärre, currently Chairman of the Research Board at the Swedish Cancer Society and Member of the Nobel Committee of the Nobel Assembly of Karolinska Institutet. In the middle, the author of this paper. Several reflections come to mind watching the figure. It was a tight and highly interacting environment. We all shared the same office (actually, we only had half the office since we shared it with another group). Clearly, the office was far from a paperless. Noteworthy, computers had not moved into our office desks. The department had, however, moved on from having only one central computer in the early‐1980s (operating almost like a core‐facility—only a few scientists know how to use it themselves) towards several ones including individual computers in the late 1980s

How NK cells might be triggered by target cells lacking self‐specific H‐2 (mouse MHC class I) molecules became my first undergraduate project, and later on formed the basis for my PhD thesis. Under Klas Kärre's supervision, we started off with NK cell‐resistant murine lymphoma cell lines which expressed high levels of “self” H‐2^b^‐molecules on their cell surface. These “wild‐type” cells were treated with a chemical mutagen and several rounds of anti‐H‐2^b^ antibodies and complement, which selected for mutant cell lines that lacked H‐2 molecules.^9^, ^11^ When the mutant cell lines were tested for susceptibility to NK cell lysis it was found, very much to our excitement, that they were sensitive to NK cell lysis. Furthermore, in contrast to the wild‐type cell lines, the mutant cell lines were rejected when inoculated into immunocompetent syngeneic C57BL/6 (B6) mice, and the in vivo rejection responses were found to be NK cell‐dependent.^9^, ^11^, ^12^, ^13^ When MHC class I expression was later restored in these mutants (which was an extensive effort at the time), the mutant cell lines regained the NK cell‐resistant phenotype, providing direct evidence for Klas Kärre's original hypothesis.^14^, ^15^, ^16^, ^17^ In parallel, fellow graduate students Petter Höglund and the late Claes Öhlén demonstrated that NK cell recognition of target cells could not only be caused by the elimination of specific MHC class I molecules on target cells. Rejection of wild‐type (H‐2^b^) lymphoma cells and bone marrow grafts could also be observed upon introduction of an MHC class I transgene (here H‐2D^d^) in B6 mice^18^, ^19^, ^20^ (Figure 1). In a review published in 1990, we coined the term “missing‐self” recognition to describe the NK cell‐mediated rejection response against target cells lacking expression of one or more self‐MHC class I molecules.^21^ In retrospect, describing the observed rejection responses with a “catchy name” turned out to be valuable for the field. The name is still frequently used in textbooks and scientific publications to describe the phenomena of NK cell recognition of target cells lacking expression of self‐MHC class I molecules.

## 3 ADDITIONAL SUPPORT FOR “MISSING‐SELF” FROM STUDIES OF MHC CLASS I‐DEFICIENT MICE

Additional research by many others subsequently confirmed that mouse MHC class I molecules interfered with NK cell recognition of target cells. Important contributions came not the least from studies of beta_2_microglobulin (β2m)‐deficient mice, which were defective in the expression of MHC class I molecules on their cells’ surface. The availability of these mice made it possible to study NK cell recognition of normal untransformed cells lacking expression of MHC class I molecules. Primary cells from these mice were found to be susceptible to lysis by B6 (wild‐type) derived NK cells, and MHC class I‐deficient bone marrow grafts from these mice were rejected by wild‐type mice in vivo.^22^, ^23^, ^24^ Similar results were later confirmed in studies of TAP‐1 mutant mice.^25^ Studies of β2m‐deficient mice and other related MHC class I‐modified mice also made it possible to study NK cell development, receptor expression, and function in relation to host MHC class I expression.^26^, ^27^, ^28^ In the course of these early studies, an interesting observation was that NK cells in β2m‐deficient mice were themselves tolerant towards β2m‐deficient target cells, a phenomenon first explained several years later. Strikingly, NK cells need to be “licenced”/“educated” by interaction with self MHC class I molecules providing them full functionality, including the ability to recognize target cells expressing “missing‐self”.^29^, ^30^, ^31^, ^32^ Recently, we have learnt that this gain in functionality involves distinct compartmentalization of activating and inhibitory receptors in licenced and unlicensed NK cells and lysosomal remodelling.^33^, ^34^

## 4 FIRST INSIGHTS INTO MHC CLASS I‐SPECIFIC INHIBITORY AND ACTIVATION RECEPTORS

**FIGURE 2:**
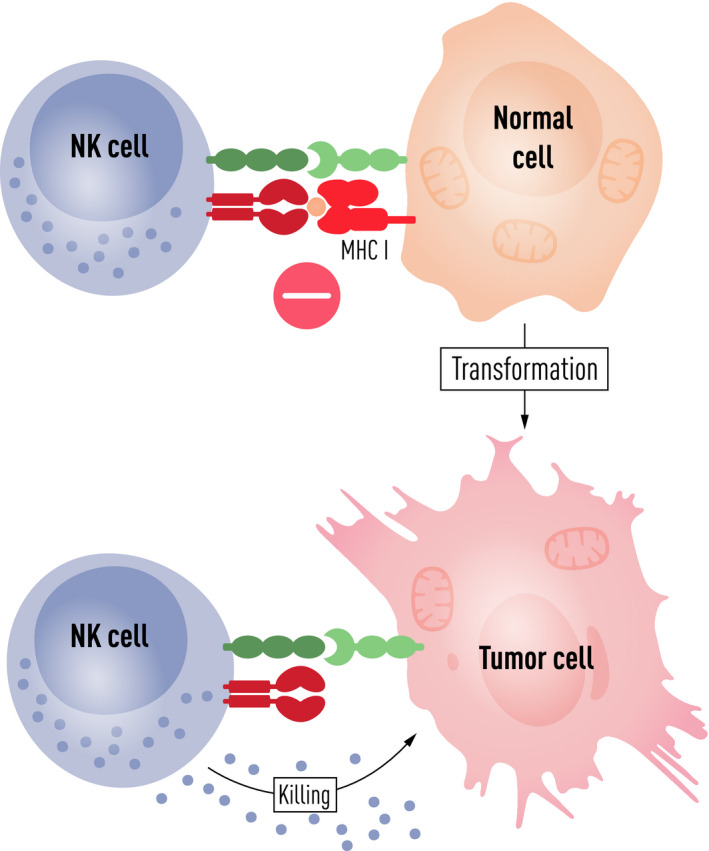
NK cell‐mediated “missing‐self” recognition of tumour cells. Towards the millennium shift, it had become clear that NK cell activation was regulated by a balance between signals mediated through activating (green) and inhibitory (red) receptors. In many situations, inhibition dominated over activation (upper part of the figure). Upon cellular transformation, MHC class I molecules, ligands for inhibitory receptors, are often reduced or lost (depicted in the figure). In parallel, cellular stress and DNA damage often lead to the upregulation of ligands for activating NK cell receptors on tumour cells. Either of these events together, or one of them, will shift the balance towards NK cell activation and induction of cytolytic effector functions resulting in tumour cell killing as illustrated (lower part of the figure)

The initial studies of NK cell recognition of MHC class I‐deficient target cells were followed by the seminal discoveries of the existence of MHC class I‐specific inhibitory receptors.^35^, ^36^ Monoclonal antibodies generated against an NK cell‐expressed molecule called Ly‐49a led to killing of MHC class I‐expressing target cells by certain NK cells. This observation was first made by Wayne Yokoyama and collaborators in murine models,^35^ and soon after similar observations with other antibodies were made in the human system by the late Moretta et al^37^ These discoveries triggered a huge search for, and mapping of, specific NK cell‐inhibitory receptors by several independent laboratories.^38^, ^39^ The identification of inhibitory receptors provided one key molecular mechanism used by NK cells to identify MHC class I‐deficient tumour cells. Not unexpectedly, however, sensing the absence of self‐MHC class I molecules by NK cells was not sufficient by itself to cause target cell sensitivity. NK cells also needed direct stimulation by specific ligands to trigger activation via specific activation receptors. The identity of the latter remained elusive until some years after the discovery of inhibitory receptors.^40^, ^41^, ^42^ A pretty clear picture on NK cell recognition of tumour target cells had emerged soon after year 2000. NK cell recognition and effector functions were governed by fine interactions involving integration of signals from both activation and inhibitory receptors (Figure 2), where inhibition often dominated over activation stimulus unless the latter was very strong. In context of today's CAR‐NK cells, this represents a situation where activation may readily override inhibition despite expression of ligands for inhibitory receptors on target cells.^43^

## 5 CONTRIBUTION TO THE INSIGHTS ON NK CELLS INHIBITORY AND ACTIVATION RECEPTORS

During the late 1990s and towards the millennium shift, several projects were going on in our laboratory providing insights into NK cell recognition of target cells. A few recollections of work conducted in our laboratory are mentioned here. To verify the ability of NK cell‐inhibitory receptors to directly recognize MHC class I molecules, graduate student Kambayashi et al^44^ demonstrated that purified MHC class I molecules could inhibit activated NK cells in a cell‐free system. Salcedo et al^45^, ^46^ contributed indirectly to the discovery of NKG2A receptor on mouse NK cells by demonstrating that the Qa‐1b molecule (mouse HLA‐E counterpart) bound to a large subpopulation of murine NK cells. Wilson, Chambers and others studied the intriguing triggering of NK cells via the CD40, CD80 and CD86 receptors.^47^, ^48^, ^49^ These interactions played an important role in the interpretation of outcome of NK cell interactions with dendritic cells,^50^ the latter a phenomenon that Chambers and Wilson were among the first to recognize and which more recently have received significant interest.^51^, ^52^ Another research focus was on 2B4‐CD48 interactions, particularly in the context of NK cell control of adaptive CD8 T cell responses.^53^, ^54^ An unexpected finding at the time was the finding that NK cell‐related receptors, including the hallmark mouse NK cell receptor NK1.1, were found to be expressed on activated CD8 T cells.^55^ Finally, together with Krister Kristersson, a well‐known neuroscientist, and his student Backström, our group characterized NK cell‐mediated interactions with dorsal root ganglia neurons,^56^, ^57^ While perhaps of only moderate interest at the time, these studies have recently (20 years later) generated renewed interest when it was found that specific autologous NK cell‐based immunotherapies triggered herpes zoster activation following infusion (Nahi et al, to be published). As it sometimes happens, older preclinical results suddenly become relevant to recent clinical observations.

## 6 FURTHER DISSECTION OF HUMAN NK CELL ACTIVATION

Our early work provided some of the first key insights into the molecular specificity of NK cells, and subsequent studies by many laboratories led the identification of the multitude of activating and inhibitory NK cell receptors. After year 2000, we switched our research effort in murine model systems towards studying human NK cells. Initial studies included fine‐mapping of the minimal requirements for induction of cytotoxicity by resting NK cells. In collaboration with Eric Long, Yenan Bryceson, a graduate student in our group, set‐up a reductionist system to fine‐map the control of NK cell cytolytic granule polarization and degranulation by different receptors in resting NK cells.^58^ These events were found to be controlled by different receptor‐ligand interactions. Subsequent studies led to the identification of synergies among specific receptors in the activation of NK cell natural cytotoxicity and cytokine secretion^59^; see also.^60^, ^61^ More recently, Bryceson among many other studies provided a detailed comparison of primary human cytotoxic T cell and NK cell responses, which revealed striking similarities in molecular requirements for lytic granule exocytosis.^62^ Many of the insights generated in these studies are valuable for today's NK cell‐based cancer immunotherapy, as discussed further below.

## 7 INSIGHTS INTO HUMAN NK CELL TERMINAL DIFFERENTIATION AND THE HUMAN KIR REPERTOIRE

While the inhibitory killer‐cell immunoglobulin‐like receptors (KIR) and NKG2A receptors had been described early on, around 2005 little was still known about their expression on a population level, in the NK cell population of a single individual, as well as their expression patterns in different individuals. Of relevance for allogeneic HSCT and allogeneic NK cell‐based immunotherapies, it became of importance to address questions about the KIR repertoires expressed by human NK cells and to provide accurate estimates of the size of the alloreactive repertoire in donors; studies that were pioneered in our laboratory by Cyril Fauriat and Kalle Malmberg.^63^ Subsequent studies addressed KIR acquisition during the terminal differentiation of human NK cells and its role in relation to self‐HLA class I ligands.^64^ Further insights into the terminal differentiation of human NK cells, including the introduction of assessments of CD57 expression, were revealed by graduate student Niklas Björkström in around 2010. He characterized the process of CD56^dim^ NK cell differentiation based on the expression of NKG2A, KIR and CD57.^65^ Additional fine‐resolution expression studies of the KIR repertoire were conducted by Malmberg and Béziat, including studies of HCMV infection and its imprint on the KIR receptor repertoire.^66^, ^67^ Many of these, and other related, studies have provided valuable insights into the development of allogenic NK cell‐based immunotherapies for human cancer.

## 8 INSIGHTS INTO SPECIFIC NK CELL SUBSETS INCLUDING “ADAPTIVE” NK CELLS

Prior to the studied outlined above, it was known that CD56^bright^ and CD56^dim^ NK cells constitute main groups of differentiated NK cells, CD56^bright^ NK cells being more prominent in tissues and CD56^dim^ NK cells more prominent in peripheral blood. In a more detailed assessment of their overall function, Fauriat and Bryceson confirmed CD56^bright^ NK cells being profound cytokine producers upon cytokine‐stimulation, while CDCD56^dim^ NK cells being both prominent cytokine producers and cytotoxic cells predominantly triggered by target cell stimulation.^68^ While these subsets constitute most of the studies concerning human NK cells, studies on a population of CD56 negative (CD56^neg^) NK cells occasionally appear in the literature. Rather little has been known with respect to these cells in relation to other NK cell subsets including those mentioned above.^69^ In collaboration with Johan Sandberg, Lothar Jänsch et al, we hence concluded an extensive proteomic analysis of these cells, revealing striking similarities between CD56^neg^ cells and CD56^dim^ NK cells, as well as a few distinct characteristics.^70^ Finally, some 10 years ago, studies on the phenomenon of NK cell “memory”^71^ and in particular so called “adaptive” NK cells caught significant interest.^72^ Adaptive human NK cells largely constitute terminally differentiated CD56^dim^ NK cells. The studies emerged from studies of the NKG2C receptor, an activating NK cell receptor that recognizes HLA‐E on targets. In this context, Miguel López‐Botet and collaborators observed increased numbers of NKG2C‐positive NK cells in individuals infected with HCMV.^73^, ^74^ These NK cells were among others characterized in detail by Schlums and Bryceson,^75^ and in parallel also by Sun, Lanier et al.^76^

In their studies, CMV was shown to drive an epigenetic diversification of NK cells, leading to altered signalling and effector functions. It is now well known that adaptive NK cells constitute a subset of highly differentiated NK cells that expands naturally in vivo in response to HCMV infection, carries unique repertoires of inhibitory KIR and displays strong cytotoxicity against tumour cells.^66^ Further studies have shown that NKG2C have the possibility to recognize polymorphic CMV peptides.^77^ In this context, it has been noted that about 4% of all humans carry a homozygous deletion of NKG2C. In these individuals, a critical role for CD2 was identified as a candidate receptor likely driving signals necessary for adaptive NK cell responses.^78^

## 9 INSIGHTS INTO TISSUE‐SPECIFIC NK CELLS

For a long time being a rather small area of research,^79^ there has more recently been an increasing interest in studies of organ‐specific NK cells. It is now accepted that NK cells circulating in the blood might not always mimic similar cells in peripheral tissues. Tissue‐specific traits of NK cells may relate to their developmental origin, tissue localization, and/or imprints caused by other factors such as viral infections.^80^ Within our own research environment, Nicole Marquardt, Jakob Michaelsson, and Niklas Björkström and colleagues have over the last ten years made significant contributions towards the characterization of tissue‐specific NK cells, including studies of NK cells in human liver and uterus,^81^, ^82^ subsequently followed by extensive studies in the human lung.^83^, ^84^ The latter studies were facilitated by the ability to trace specific subpopulations of cells expressing tissue‐specific markers such as CD69, CD103 and CD49a, and other specific molecular signatures. Much more is clearly to be learnt within this field of research, including much needed studies on tumour resident NK cells. The latter not the least important in relation to cancer immunotherapy.

## 10 PRIMARY IMMUNODEFICIENCIES AFFECTING NK CELLS

Primary immunodeficiencies affecting NK cells have severe consequences, particularly regarding susceptibility to viral infection.^85^, ^86^, ^87^ Following his successful studies on the minimal requirements for activation of human NK cells, Yenan Bryceson performed an in‐depth exploration of primary immunodeficiencies that affect NK cell (and other lymphocyte) cytotoxicity. He early on described how defective expression of syntaxin‐11 led to impaired degranulation, providing additional insights into the molecular basis for familial FHL type 4.^88^ In a similar manner, FHL‐3 was demonstrated to be caused by a deep intronic mutation and inversion in UNC13D.^89^ Other studies of NK cell activation demonstrated how different NK cell‐activating receptors could contribute to the recruitment of Rab27a and/or Munc13‐4 to perforin‐containing granules for induction of cytotoxicity.^90^ While these and related studies demonstrated dramatic, often life‐threatening effects of NK cell deficiencies upon viral infection, more subtle effects may also prime for other diseases, including cancer.^91^ Several more recent studies by Voss and Bryceson^92^ have provided detailed insights into human NK cell deficiencies. In this context, we have hypothesized whether the adoptive transfer of intact NK cells could halt an ongoing inflammatory syndrome caused by an inability of NK cells to clear the body from activated T cells and macrophages.

## 11 NK CELL IN VIRAL INFECTIONS

As revealed from studies of not the least primary immunodeficiencies, NK cells play an important role in host responses to many virus infections. This is an area of research in which we rather extensively have been involved in over the years. Here, only a few select studies are discussed, since the primary focus on this review is oriented around tumour cell recognition. Niklas Björkström pioneered studies of NK cells in human hantavirus infection. These studies provided insights into their ability to rapidly respond to an acute infection,^93^ and into mechanisms underlying sustained activation‐related responses.^94^ In studies of hepatitis C and hepatitis delta virus infections, antiviral treatment interventions have demonstrated effects on NK cells that correlate with disease outcomes.^95^, ^96^ Other studies have noted chronic compromised NK cell function in both acute and chronic hepatitis^97^ and a seemingly irreversible impact on the diversity of the NK cell repertoire following successful disease treatment.^97^ NK cell activation has also been studied in acute flavivirus infection including tick‐borne encephalitis and Dengue virus infection.^98^, ^99^ In the case of Dengue virus infection, activated NK cells notably show priming of homing to sites of infection as demonstrated by Björkström et al^99^ Most recently, we have in a concerted action conducted the first study on human NK cell activation in the course of clinical SARS‐CoV‐2 infection.^100^ Many of these studies provide information on the inherent NK cell responsiveness to the viral infection. It is interesting to note viral infection are often brought up in context of adoptive NK cell‐based immunotherapies, including the current ongoing SARS‐CoV‐2 virus pandemic.^101^ While NK cells could contribute to antiviral host responses, it is, however, an open question as to if they may also contribute towards driving harmful inflammatory responses.^100^

## 12 NK CELLS IN ALLERGY AND AUTOIMMUNITY

Although NK cells are best characterized in terms of their ability to control infections and tumours, recent data have indicated that they also are important regulatory cells due to their interactions with many types of immune and nonimmune cells. They can thereby affect the outcome of adaptive immune responses and maintain immune homeostasis. NK cells can either exacerbate or limit immune responses, including those to autoantigens and/or allergens. Shi, a talented post doc in our group, early on studied NK cells in several autoimmune models, including experimental autoimmune myasthenia gravis.^102^, ^103^ In one of his studies, NK cells were found to participate in the development of myasthenia gravis (a T cell‐dependent, B cell‐mediated and antibody‐mediated autoimmune disease) in B6 mice. The requirement for NK cells was reflected by the lack of a type I helper T cell response and antibodies to the acetylcholine receptor in both NK1.1^+^ cell‐depleted and NK cell‐deficient IL‐18−/− mice.^104^, ^105^ In parallel, in collaboration with Magnus Korsgren and colleagues, we also early on investigated allergen‐induced eosinophilic airway inflammation in mice. The study suggested a critical role for NK cells in development of allergen‐induced airway inflammation and that this effect of NK cells is exerted during the immunization.^106^ Compared to studies in cancer or viral infection, studies of NK cells in context of autoimmunity and allergy clearly need much more exploration.

## 13 NK CELLS IN CANCER

As described in the introduction of this review, NK cells were discovered because of their ability to specifically lyse tumour cells without prior sensitization. Many studies over the years have confirmed the ability of NK cells to prevent the development of cancer and to avoid relapse following adoptive cancer immunotherapy.^107^, ^108^, ^109^ We now have extensive knowledge of the molecular basis for NK cell recognition of human tumour cells. We also have ample evidence for NK cell‐mediated ex vivo killing of human primary tumour cells.^110^ We know that some NK cells have the capacity to migrate to, and reside in, the human tumour microenvironment.^111112^ Individuals with high levels of NK cell cytotoxicity have provided indirect evidence for NK cell immunosurveillance of human cancer due to their reduced incidence of cancer.^113^ As will be discussed further on, marked clinical responses have been observed following infusion of human NK cells into cancer patients and insights into the molecular nature of responses observed have been gained.^114^ These observations all indicate, either directly or indirectly, a role for NK cells in mediating cancer immunosurveillance.^107^ As we shall see towards the end of this review, this opens up exciting possibilities for exploration of NK cells in context of adoptive immunotherapy.

## 14 RECOGNITION OF PRIMARY TUMOUR CELLS BY NK CELLS

As outlined in a review some years ago,^110^ studies on the interactions between NK cells and freshly isolated human tumour cells are comparably relatively scarce compared to studies conducted on cell lines. In some cases, experiments are hampered by technical challenges, including how to monitor specifically the lysis of fresh tumour cells within heterogeneous patient‐derived cell populations. Some efforts have been made in our constellation in this respect. With respect to solid tumours, Carlsten studied NK cell interactions towards human ovarian carcinoma cells.^115^ With respect to haematological tumours, Alici studied NK cell‐mediated interactions with isolated multiple myeloma cells,^116^ suggesting that several activating receptors might contribute to lysis of multiple myeloma cells. In related studies, Malmberg, Carlsten et al addressed the impact of host tumour cells on NK cell receptor expression in cancer patients.^117^, ^118^ Downregulation of select NK cell receptors, including DNAM‐1, was observed. In an another set‐up, Malmberg and Liu studied ex vivo‐expanded adaptive NK cell.^119^ These cells were found to be specific and highly efficient killers of allogeneic pediatric T and precursor B cell acute lymphoblastic leukaemia blasts, previously shown to be refractory to killing by autologous NK cells and the NK cell line NK92. Interestingly, selective expansion of NK cells that express one single inhibitory KIR for self‐HLA class I (a common trait for many adaptive NK cells) could allow exploitation of the full potential of NK cell alloreactivity in cancer immunotherapy.^120^

## 15 PROSPECTS FOR USING NK CELLS IN HUMAN CANCER IMMUNOTHERAPY

The gained insights into the molecular specificities that regulate NK cell function suggested that it should be possible to design NK cell‐based immunotherapeutic strategies to combat malignant diseases. The very first NK cell‐based clinical study in our own laboratory was undertaken by Sirac Dilber and Evren Alici, based on earlier development of expansion protocols for clinical‐grade NK cells and analyses of these cells.^116^, ^121^, ^122^ The safety of donor‐derived long‐term ex vivo‐expanded human NK cells was evaluated when given as donor lymphocyte infusions in patients with solid tumours following allogeneic HSCT. Infusion of the cells was found to be safe whether administered alone or with IL‐2 subcutaneously. No signs of acute GvHD were observed. Interestingly, in the clinical study, one patient with hepatocellular carcinoma showed markedly decreased serum alpha‐fetoprotein levels following cell infusions.^123^ These early findings suggested that the use of the present ex vivo‐expanded NK cells was safe, and that NK cells might constitute an attractive approach for further clinical evaluation in cancer patients. The study served very much to spark our interest in exploring NK cells in different therapeutic settings in the years to come.

## 16 ALLOGENEIC NK CELL‐MEDIATED IMMUNOTHERAPY IN HIGH‐RISK MYELOID MALIGANANCIES

In parallel with the studies above, an interest in exploring the possibility of using allogeneic NK cells in context of human cancer immunotherapy had developed in our group.^120^, ^124^ The interest was inspired by studies conducted by Ruggeri and collaborators in context of donor NK cell alloreactivity in mismatched haematopoietic transplants,^125^ as well as on the pioneering studies in the field of allogenic NK cell‐based immunotherapies by Jeffery Miller and collaborators at the University of Minnesota.^126^ Our own efforts were led Andreas Björklund, Kalle Malmberg and collaborators who set out to test the safety, efficacy, and immunobiological correlates of allogeneic NK cells in primary chemotherapy‐refractory or relapsed high‐risk myelodysplastic syndrome (MDS), secondary AML (MDS/AML), and de novo AML patients. In their initial study, sixteen patients received fludarabine/cyclophosphamide conditioning combined with total lymphoid irradiation followed by adoptive immunotherapy with IL‐2‐activated haploidentical NK cells.^127^ NK cell infusions were well‐tolerated, with only transient adverse events observed. Notably, six patients achieved objective responses with complete remission (CR), marrow CR or partial remission. Five patients proceeded to allogeneic HSCT. Three patients were still free from disease >6 years after treatment.^127^ Overall, this study suggested that high‐risk MDS and AML are responsive to NK cell therapy and supported the use of haploidentical NK cell infusions as a bridge to HSCT in refractory patients. These studies now form the basis for the development of novel allogeneic NK cell‐based immunotherapy protocols in our laboratory.

## 17 AUTOLOGOUS NK CELL‐MEDIATED IMMUNOTHERAPY IN MULTIPLE MYELOMA

While a strong rational for allogeneic NK cell‐based trials has been emphasized internationally and also been supported by our own studies,^127^ autologous treatment strategies also present a possibility. Based on this idea, Evren Alici, Hareth Nahi and collaborators decided in parallel with the allogenic NK cell‐based trial described above to conduct an autologous phase I/II clinical trial in which multiple myeloma patients had undergone upfront autologous haematopoetic stem cell transplantation (HSCT). In the study, patients were infused with multiple doses of ex vivo activated and expanded autologous NK cells. This strategy aimed to diminish MRD and to ultimately delay or prevent tumour progression. A reduction of the M‐component and/or deepening of minimal residual disease was observed. Exploratory analysis of peripheral blood and bone marrow aspirates revealed increased numbers of infused NK cells in peripheral blood following treatment and notably marked elevations in plasma Granzyme B‐levels (a correlate of cytotoxic responses) following each consecutive autologous NK cell infusion. Strikingly, increased levels of Granzyme B were detected in bone marrow aspirates four weeks after the last infusion of autologous NK cells (Nahi, Alici, Ljunggren et al, to be published). As we discuss below, these observations suggest possibility for further exploration of autologous NK cell‐based immunotherapies, in particular in settings where allogeneic settings are not eligible.

## 18 CLINICAL IMPLICATIONS OF ALLOGENEIC AND AUTOLOGOUS NK CELL‐BASED IMMUNOTHERAPIES

In recent years, many trials of allogeneic NK cell‐based immunotherapy have been conducted.^114^, ^128^, ^129^, ^130^, ^131^ Allogeneic NK cells have the advantage that they can be harvested for immunotherapy purposes from healthy donors, most often from peripheral blood. Current protocols also allow use NK cells from other sources, for example cord blood.^132^, ^133^ NK cells can additionally be derived from pluripotent embryonic stem cells and via iPSC technology.^134^, ^135^ Allogeneic cells clearly provide several therapeutic advantages, such as the utilization of “missing self” reactivity,^136^ and the ability to generate cells with other desired specificities; for example allowing specific expansion of “adaptive” NK cells.^119^ Finally, and most importantly, allogeneic NK cells provide the basis for the development of several types of “off‐the‐shelf” products, for which there is currently significant academic and industrial interest. Use of allogeneic NK cells, however, normally requires lymphodepleting (immunosuppressive) conditioning, hampering its use in some settings when this type of conditioning cannot be motivated from a clinical standpoint.

In contrast to what is outlined above, few studies currently rely on autologous NK cells.^137^ While the concept of using these cells is not new, studies in patients with advanced treatment‐refractory solid tumours have been met with limited success.^138^ However, in contrast to many current protocols with allogenic NK cells, autologous NK cells allow infusion without the need for lymphodepletion. Moreover, repetitive dosage is not generally a problem. Due to these and other considerations, we have speculated that these could be specifically useful in particular clinical settings, including those where allogeneic NK cells would not be applicable; for example in settings of consolidation or maintenance therapy where immunosuppressive conditioning could not easily be done or in settings of possible/suspected metastatic spread following surgical removal of the primary tumours (Nahi, Alici, Ljunggren et al, to be published). We predict that autologous NK cells could in the future allow for additional therapies beyond what is currently feasible for allogeneic NK cells.

## 19 NK CELLS IN THE ERA OF CANCER IMMUNOTHERAPY

As described above, cancer immunotherapy, including many cell‐based therapies, is currently emerging as central treatment modalities in a wide range of cancer types. Clinical examples from our own laboratory described here illustrate that NK cells can contribute to the control of human cancer via adaptive immunotherapy. Clearly, the field is only in its beginning, and we foresee numerous immediate and long‐term developments with respect to NK cell‐product development and design of clinical protocols. Several other developments may furthermore contribute to the field of NK cell‐based immunotherapies. NK cell‐specific checkpoints, including antibodies directed against inhibitory KIRs and NKG2A as well as PD‐1/PD‐L1, are currently in late‐stage clinical development and may under certain circumstances unleash the cytotoxic potential of the patient's own NK cells.^139^, ^140^ Understanding the cellular and molecular mechanisms that regulate effector function, homeostasis and homing of human NK cells may pave the way for improving current NK cell‐based products and improve clinical efficacy. Another important area of research is to further explore combinations of immunomodulatory drugs and therapeutic antibodies together with NK cell‐based immunotherapies.

## 20 INDUSTRIAL DEVELOPMENT OF NK CELL‐BASED PRODUCTS

NK cells are now suggested to “be poised” to become key components of multipronged therapeutic strategies for human cancer.^114^ Significant expansion with respect to development of NK cell‐based immunotherapies in small and medium enterprises (SME) have been observed the last years. Recently, we have also witnessed several collaborative interactions, mergers, and/or of other forms of agreements between several SMEs and large Pharma in the field of NK cell‐based immunotherapies. Specifically dedicated scientific meetings such as, for example the recently held Innate Killer Summit 2020 (www.innate‐killer.com), currently draw a significant audience from academia, SME and the Pharma industry. Themes at the most recent Innate Killer Summit meeting included discussions on the potential of genetically engineered, off‐the‐shelf, and cost‐effective solutions to optimize results and strategies towards getting to the clinical faster in the space of NK cell‐based immunotherapies. Key development areas discussed included enhanced manufacturing and process development, the exploitation of the potential of the iPSC technology, and strategies towards targeting solid tumours through genetic modification. The holy grail in several contexts is the strive towards generating “universal cells”; that is allogeneic cells being able to escape host immune responses. Taken together, through current academic and industrial developments we foresee that NK cells are poised to become key components in future therapeutic strategies against human cancer. Clearly a striking development from the very first early studies in the mouse, almost 50 years ago.

## 21 LESSONS LEARNT AND EXPERIENCES GAINED

The present review encompasses some personal recollections and reflections of research work conducted in our laboratory over three decades, leading up towards the utilization of NK cells in the context of human cancer immunotherapy. Throughout the years, many other areas of research have also engaged our group. Early on, we stumbled upon findings that led to the discovery of the class I pathway for antigen processing and presentation and MHC class I peptide binding.^141^, ^142^ This in turn led to an interest in studying many aspects of T cell biology and function, most recently in COVID‐19.^143^ Although much of our research over the years has focussed on studies relating to cancer, we have maintained a parallel interest in studies of viral infections. Our most recent studies of NK cells, again in context of COVID‐19, is one example of this.^100^ Reflecting upon this, work over the years in different but related research areas likely catalysed our thinking and enhanced our overall knowledge of benefit for our present studies relating to NK cells, and in particular the development of NK cell‐mediated immunotherapies for human cancer and possibly also some viral diseases. Shifting our research from studies in murine experimental towards studies of human NK cells around the millennium shift was a strategic move and turned out to be very valuable. It provided us with novel opportunities in exploring NK cells in context of human health and disease, and development of novel treatment strategies to deal with disease conditions such as human malignancies. The latter move turned out not only to be valuable with respect to our own academic interest, but it also to the increasing interest in human immunity in general evolving in parallel with development at the time as well as the development of new technologies (eg, advanced flow cytometry, RNA‐Seq technology and systems analysis), all significantly facilitating studies of the human immune system. Finally, I'm grateful having had the privalage of being trained under outstanding scientific leaders, particularly Klas Kärre and Hidde L. Ploegh. The value of this can not be underestimated. Furthermore, I have been lucky throughout the years being able to work with fantastic graduate students, post docs, and collabotators. Without the latter, we would not have been where are are today.

## CONFLICT OF INTEREST

With respect to NK cell‐based immunotherapy, I am a co‐founder and board member of Vycellix Inc and XNK Therapeutics AB. I serve as a scientific advisory board member for Morphogenesis, Hope Bio‐Sciences/TRIGR and XNK Therapeutics. I have royalty from FATE Therapeutics. I serve as the Director of the NextGenNK Competence Center, supported by Sweden's Innovation Agency under the Ministry for Enterprise and Innovation, with the task to foster interactions and collaborations between SME/Industry, academia, and society. Finally, with respect to the Scandinavian Journal of Immunology, I have served on its Editorial Board (1996‐2004), as its Deputy Editor‐in‐Chief (2004‐2018), and as one of its two Advisory Editors (2018).

## Data Availability

The present article is a review encompassing published results.
